# A review of the participation of DDIT4 in the tumor immune microenvironment through inhibiting PI3K-Akt/mTOR pathway

**DOI:** 10.3389/fonc.2025.1595463

**Published:** 2025-08-18

**Authors:** Yunshu Jiao, Yang Xiang

**Affiliations:** ^1^ National Clinical Research Center for Obstetric & Gynecologic Diseases, Department of Obstetrics and Gynecology, Peking Union Medical College Hospital, Chinese Academy of Medical Sciences & Peking Union Medical College, Beijing, China; ^2^ Clinical College, Chinese Academy of Medical Sciences and Peking Union Medical College, Beijing, China

**Keywords:** DDIT4, mTORC1, tumor immune microenvironment, autophagy, PI3K-Akt/mTOR pathway

## Abstract

DDIT4 (DNA Damage Inducible Transcript 4), a well-established inhibitor of the PI3K-Akt/mTOR pathway, is upregulated under cellular stress conditions. Extensive research has demonstrated that DDIT4 expression is aberrantly elevated in various malignancies, where it exhibits context-dependent roles in either tumor promotion or suppression. However, the mechanisms underlying how DDIT4 is involved in tumor immune regulation remain to be fully elucidated. This review systematically summarizes the multifaceted mechanisms by which DDIT4 participates in tumor immunomodulation, primarily through its inhibition of the PI3K-Akt/mTOR pathway to induce autophagy activation and metabolic reprogramming; furthermore, it comprehensively examines DDIT4’s regulatory effects on various components within the tumor immune microenvironment, including tumor cells, both innate and adaptive immune cells, and immunomodulatory cytokines. This comprehensive analysis aims to establish a theoretical foundation for considering DDIT4 as a potential therapeutic target in tumor immunotherapy.

## Introduction

Gene DNA damage inducible transcript 4 (*DDIT4*) is pervasively expressed at low levels in most adult tissues (1), while its expression increases under the induction of multiple cellular stresses like hypoxia (2), energy depletion (3), and treatments like ionizing radiation(IR) (4) and chemotherapeutic DNA damage agent etoposide (5) and temozolomide (4). Associated with the existence of above-mentioned inducible factors in the tumor microenvironment (TME), DDIT4 is highly expressed in a variety of cancers (6). The heterogeneous role DDIT4 plays in multiple kinds of cancers has been extensively studied (**Supplementary Table S1**), indicating *DDIT4* can act as either an oncogene or a tumor suppressor gene: DDIT4 acts as a negative regulator of invasiveness in non-small cell lung cancer (NSCLC) (7), and the up-regulation of DDIT4 is an pivotal part of the apoptosis promotion of human breast cancer cells by the combination of melatonin and arsenic trioxide (8), whereas overexpression of DDIT4 is related to advanced pathological features in colorectal cancer (9), and DDIT4 stimulates proliferation and tumorigenesis in gastric cancer (10), etc. It is commonly recognized that the context-dependent role of DDIT4 is highly interdependent with the complexity of TME (11).

TME represents a highly complex and dynamic ecosystem comprising diverse cellular components, extracellular matrix constituents, vascular networks, and an array of soluble signaling molecules, including cytokines. The TME is characterized by distinct metabolic alterations, prominently featuring metabolic reprogramming with aerobic glycolysis (the Warburg effect) as the predominant energy production pathway. This metabolic shift, coupled with the development of hypoxic conditions, play crucial roles in tumor adaptation and progression (12). Furthermore, the tumor immune microenvironment (TIME) is fundamentally constituted by the multifaceted interplay between innate and adaptive immune cells, along with a complex network of immunomodulatory cytokines and chemokines, which collectively orchestrate critical processes including tumor immune surveillance, immune evasion mechanisms, and responses to immunotherapeutic interventions. It can be speculated that tumor immunomodulation plays an integral role in the heterogeneity of DDIT4 in different tumors (13).

DDIT4 is primarily recognized for its crucial role in suppressing mechanistic target of rapamycin complex 1 (mTORC1), a master regulator with mTOR as core subunit in PI3K-Akt/mTOR pathway (6). The PI3K-Akt/mTOR pathway is acknowledged as a nutrient-sensing mechanism that enhances cell proliferation and protein synthesis in the presence of adequate nutrition and optimal environmental factors (14). When mTORC1 is suppressed by upregulated DDIT4 expression, the consequent induction of metabolic reprogramming and autophagy activation exerts significant immunomodulatory effects within the TME (15). The DDIT4-mediated inhibition of the PI3K-Akt/mTOR pathway may modulate the TIME through multiple mechanisms, and a comprehensive understanding of these regulatory pathways could provide valuable insights for optimizing the precision and efficacy of tumor immunotherapy.

## The location and structure of DDIT4

*DDIT4*, also known as regulated in development and DNA damage response 1(*REDD1*) (16) or responsive to hypoxia 801 (*RTP801*) (17), is situated on chromosome 10 (10q22.19). According to immunofluorescence staining and confocal microscopy, DDIT4 protein is predominantly expressed in the cytoplasm and co-localized with mitochondria, which is closely related to the function of DDIT4 in regulating reactive oxygen species (ROS) production in hypoxia environment (18).

**Figure 1:**
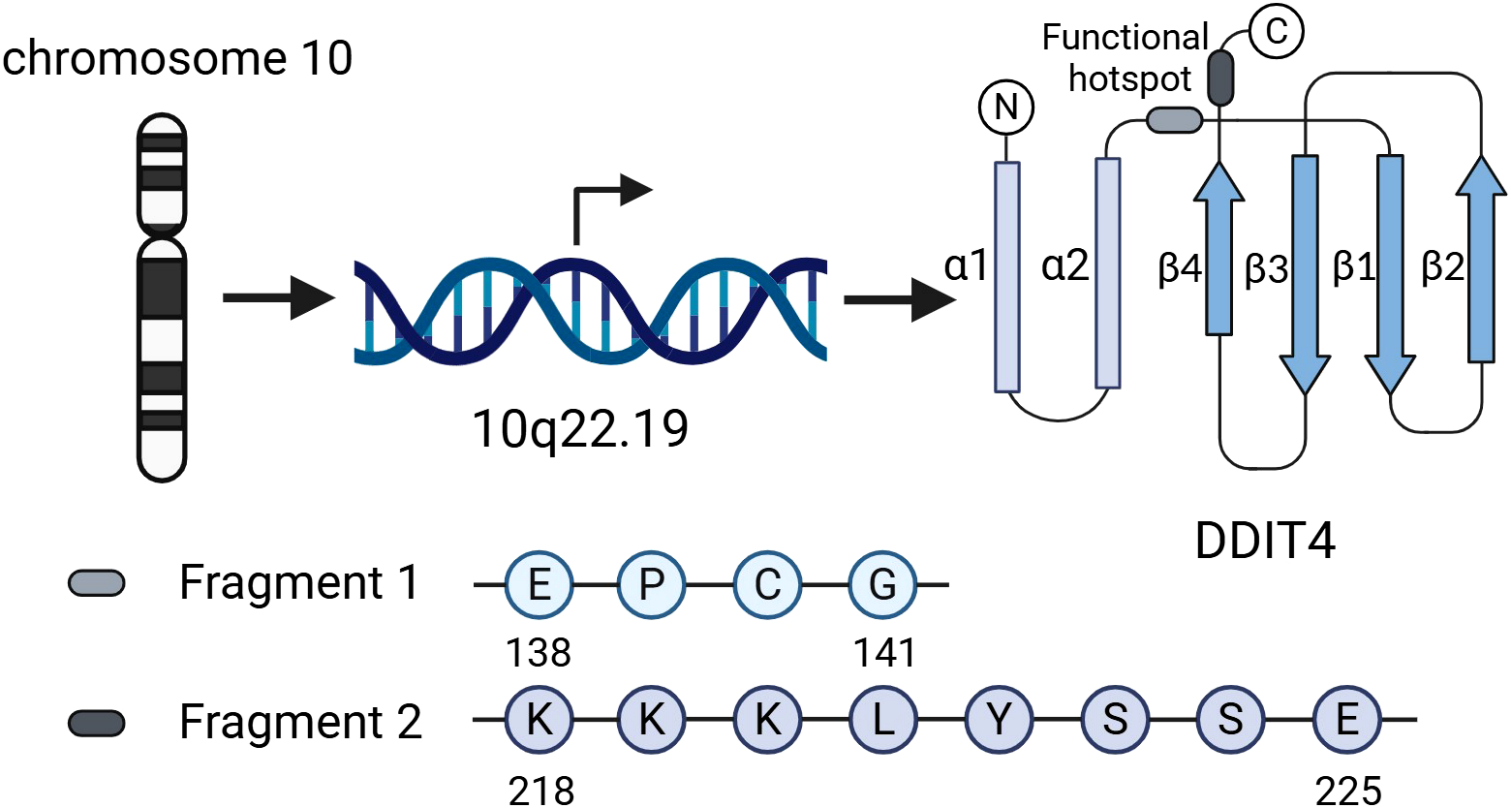
Genomic location, structural features, and functional hotspot of DDIT4. DDIT4 is located on chromosome 10 (10q22.19). Its structure adopts a two-layer α/β sandwich fold, comprising two antiparallel α-helices and a mixed β-sheet. The functional hotspot consists of two discontinuous conserved motifs (138EPCG141 and 218KKKLYSSE225), mutations in which disrupt DDIT4-mediated inhibition of mTORC1.

This overall structure of DDIT4 is characterized by a two-layer α/β sandwich consisting of two antiparallel α-helices and a mixed β-sheet, in which strands β1-β3 encompass a unique psi-loop motif (19). Researchers also found a functional hotspot containing multiple highly conserved residues in the exposed region on the surface of DDIT4 three-dimensional structure, consisting of two discontinuous sequence fragments ^138^EPCG^141^ and ^218^KKKLYSSE^225^ (**Figure 1**); mutations of the residues of this functional hotspot can abolish the inhibitory effect of DDIT4 on mTORC1 (19).

## Mechanism of DDIT4-mediated inhibition of mTORC1 complex and subsequent activation of autophagy

**Figure 2:**
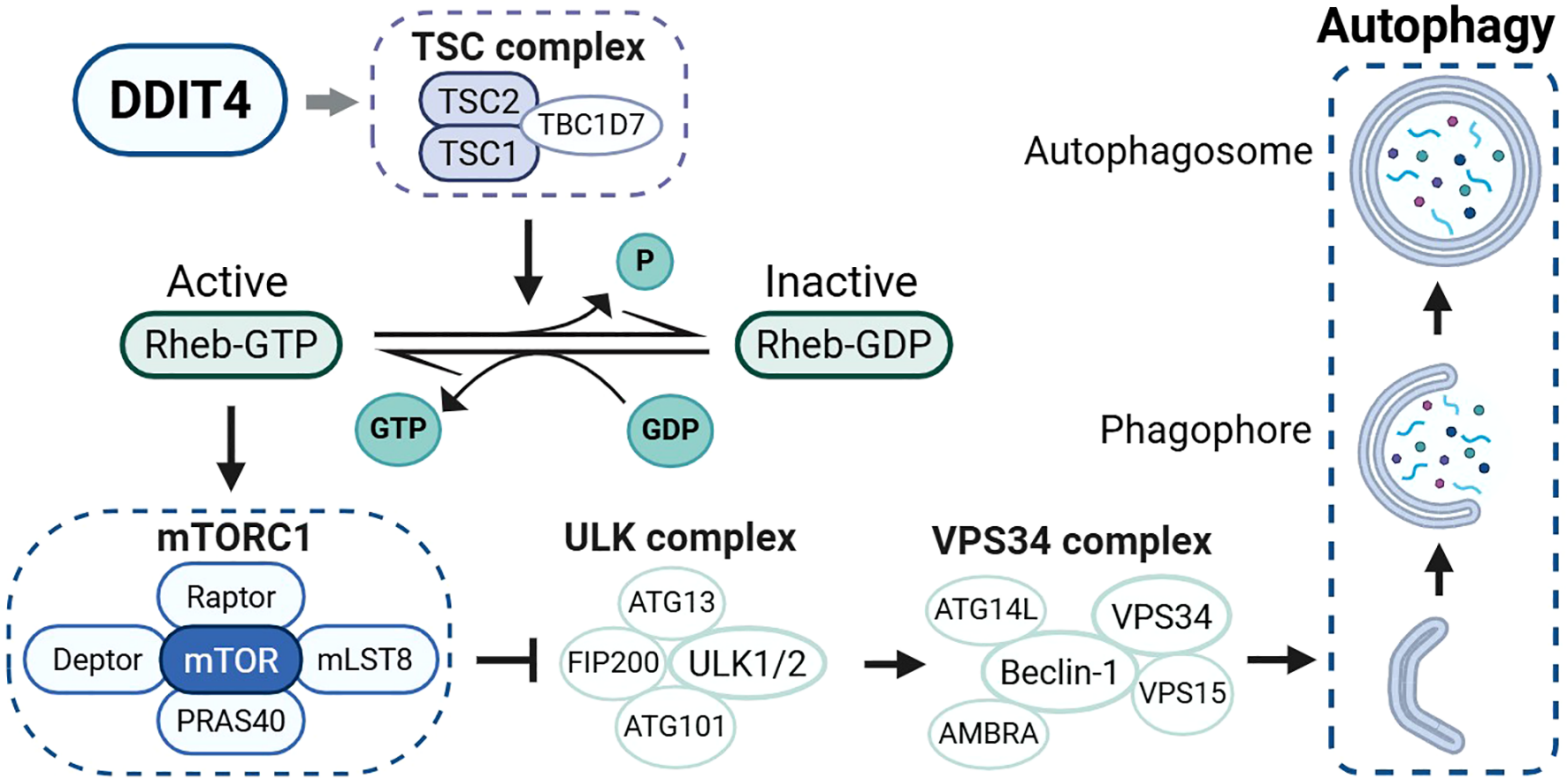
Mechanistic model of DDIT4-dependent autophagy induction through mTORC1 suppression. DDIT4 promotes TSC1/TSC2 complex assembly, which catalyzes the conversion of Rheb-GTP to Rheb-GDP, thereby inhibiting mTORC1 activity. This releases mTORC1-mediated suppression of ULK1 and VPS34 complexes, initiating autophagy formation. Solid arrows indicate activation; blunt lines show inhibition.

DDIT4 suppresses mTORC1 activity through the tuberous sclerosis complex (TSC1/TSC2); the complex does not directly interact with mTORC1 but functions as a GTPase targeting Ras homolog enriched in brain (Rheb); by catalyzing the conversion of Rheb-GTP to its inactive Rheb-GDP form, the TSC1/TSC2 complex prevents Rheb-mediated activation of mTORC1 signaling (20) (**Figure 2**).

In regard to how could DDIT4 interact with TSC1/TSC2 complex, former model proposed that DDIT4 functioned by competitively interacting with TSC2 for binding to 14-3-3 proteins; elevated expression of DDIT4 disrupts the association between 14-3-3 and TSC2, leading to the liberation of TSC2, which enables the formation of an active TSC1/TSC2 complex and the subsequent suppression of mTORC1 signaling activity (21). However, studies on the structure of DDIT4 have denied the direct interaction between DDIT4 and 14-3-3 proteins (19). Current research findings indicate that DDIT4 downregulates TSC2 phosphorylation through protein phosphatase 2A(PP2A) mediated Akt dephosphorylation, this mechanism leads to the activation of TSC2 and facilitates the assembly of the TSC1/TSC2 functional complex (22).

Under conditions of mTORC1 dephosphorylation and inhibition, its downstream signaling pathways that promote cellular proliferation and protein biosynthesis are suppressed. Concurrently, the Unc-51-like kinase (ULK) complex, an autophagy-regulating complex that is normally bound to mTORC1 and inhibited via phosphorylation at specific sites (Ser637 and Ser757 of ULK1 subunit, Ser258 of Atg13 subunit), dissociates from mTORC1 and becomes activated, thereby initiating cellular autophagy (23). Autophagy represents a fundamental cellular mechanism through which cytosolic components are sequestered and subsequently delivered to lysosomes for degradation (24). In the context of cancer, autophagy plays a dual role in tumor growth: on one hand, autophagy supports the survival and proliferation of tumor cells under nutrient-deprived conditions in the TME by degrading damaged organelles and proteins to provide essential biosynthetic precursors and energy; on the other hand, when autophagy exceeds the regulatory capacity of the cell, excessive autophagy can lead to cell death, thereby inhibiting tumor growth (25). Additionally, during tumorigenesis and progression, the interplay between autophagy and tumor immune evasion represents an intrinsic mechanism driving the advancement of malignant tumors (26).

## Regulation of tumor cell-intrinsic immune function

**Figure 3:**
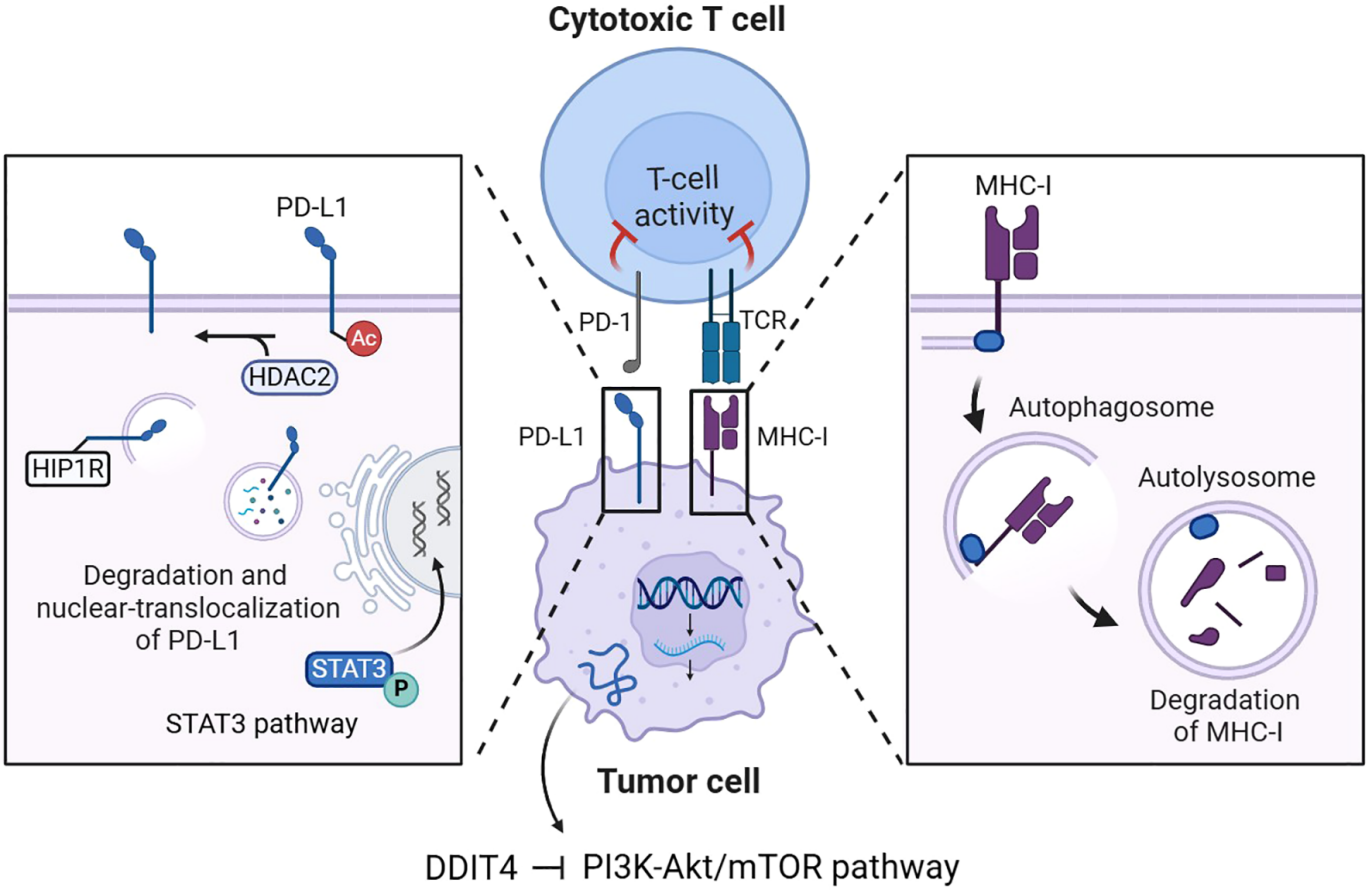
DDIT4-mediated regulation of MHC-I and immune checkpoints in tumor cells. DDIT4 inhibits the PI3K-Akt/mTOR pathway to activate autophagy in tumor cells, leading to MHC-I degradation and PD-L1 modulation through autophagic degradation, HDAC2-mediated nuclear translocation, and STAT3 transcriptional activation. These mechanisms collectively regulate cytotoxic T cell activity in the tumor immune microenvironment.

For tumor cells, common strategies of immune evasion often involve compromised antigen presentation due to mutations or loss of heterozygosity in the major histocompatibility complex class I (MHC-I), and the formation of antigen-presenting co-regulatory signals by inhibitory immune checkpoints (27). DDIT4-mediated autophagy activation through PI3K-Akt/mTOR pathway inhibition in tumor cells potentially regulates the expression of MHC-I molecules and immune checkpoint proteins, suggesting a critical link between cellular stress responses and tumor immunogenicity (**Figure 3**). Yamamoto et al. demonstrated that autophagy activation mediates MHC-I complex degradation on pancreatic ductal adenocarcinoma cell (PDAC) surfaces, a process reversible through chloroquine (CQ)-mediated autophagy inhibition (28). In another study, using *in vitro* and injection-induced tumor models of B16F10 melanoma, Py8119 triple-negative breast cancer, and MC38 colon carcinoma, researchers also found that the inactivation of autophagy associated protein 5 (Atg5) makes MHC-I-deficient tumor cells more susceptible to T cell-mediated cytokine-induced apoptosis (29). Inhibition of DDIT4-mediated mTORC1-dependent autophagy may enhance tumor antigen presentation via MHC-I, potentially promoting anti-tumor immune surveillance.

Current research has elucidated that the upregulation of inhibitory immune checkpoint molecules, such as programmed death-ligand 1 (PD-L1), on the surface of tumor cells constitutes a pivotal mechanism underlying immune tolerance. PD-L1 mediates its immunosuppressive effects predominantly through specific binding to the programmed death-1 (PD-1) receptor expressed on activated T lymphocytes, thereby inhibiting T cell proliferation and inducing T cell apoptosis. Consequently, immune checkpoint blockade (ICB) therapy has emerged as a critical class of therapeutic agents in the realm of cancer immunotherapy, offering a transformative approach to restoring anti-tumor immune responses (15).

Liao et al. verified that in the NSCLC cell lines A549 and SPC-A-1, the expression of the autophagy marker sequestosome 1 (SQSTM1, abbreviated P62) was positively correlated with PD-L1 expression (30), p62 interacts with ubiquitinated proteins and subsequently associates with microtubule-associated protein 1 light chain 3 beta-II (LC3-II) localized on the autophagosomal membrane to promote autophagy (31). Liu et al. further explained that the recognition of ubiquitinated PD-L1 by p62 and its subsequent autophagic degradation mediated by p62 could be interfered by the deubiquitinating enzyme ubiquitin-specific protease 14 (USP14) (32). These findings suggest the activated autophagy through PI3K-Akt/mTOR pathway inhibition induced by DDIT4 represents a potential mechanism of PD-L1 degradation. More specifically, the degradation of PD-L1 can be accomplished by enhancement of autophagy flux mediated by autophagy-associated proteins represented by Huntingtin interacting protein one associated protein (HIP1R) (33). The procedure also includes the involvement of histone deacetylase 2 (HDAC2), which regulates the nuclear translocation of PD-L1 by changing its acetylation level, the nuclear-translocalized PD-L1 can further transcriptionally activate the expression of multiple genes associated with immune surveillance escape (34).

Moreover, phosphorylated signal transducer and activator of transcription 3 (STAT3) can activate the transcription of oncogenes including CD274 coding PD-L1 (35), Lu et al. confirmed that impaired autophagy could induce STAT3 phosphorylation, thereby increasing PD-L1 expression (36). Combined with the above mechanisms, autophagy-mediated downregulation of tumor cell PD-L1 expression may compromise anti-PD-L1 immunotherapy efficacy by reducing target availability.

However, the impact of cellular autophagy on the expression of PD-L1 on tumor cell surfaces exhibits a non-unidirectional effect (**Supplementary Table S2**). For example, Yu et al. identified that autophagy inhibitor 3-methyladenine (3MA) could suppress PD-L1 expression, thereby augmenting the therapeutic outcomes of photodynamic therapy by enhancing the immunogenic effects in osteosarcoma mouse models (37). In another statistical analysis study based on the human triple-negative breast cancer (TNBC) cell line HS578T, the expression level of DDIT4 was negatively correlated with the levels of tumor-infiltrating immune cells and immunobiological markers, while positively correlated with the expression levels of various immune checkpoint molecules, including PD-L1 and CTLA4 (38). Beyond serving as a target for ICB therapy, sustained presence of PD-L1 on the cell surface enables tumor cells to deliver immunosuppressive signals to T cells to achieve immune escape. The bidirectional regulation of immune checkpoint molecules by mTORC1 inhibition-induced autophagy underscores the need for further mechanistic investigation.

In addition to PD-L1, autophagy was also found to play an important role in the degradation of another inhibitory immune checkpoint, Cytotoxic T-Lymphocyte-Associated Protein 4 (CTLA4). Lei et al. reconfirmed the degradation pathway of CTLA4 via autophagy-lysosome (A-L), and primarily proved that functional mTOR could facilitate the accumulation of CTLA4 by repressing the onset process of autophagy such as A-L fusion (39). These finding provide mechanistic evidence that DDIT4-mediated mTOR inhibition induces autophagy-dependent CTLA4 degradation, potentially compromising anti-CTLA4 therapy efficacy.

Based on the intricate regulatory influence of autophagy on immune checkpoint molecules, inhibition or induction of autophagy has been demonstrated to improve the efficacy of ICB therapy in multiple *in vitro* studies (**Supplementary Table S2**). Yamamoto et al. also perceived that *in vitro* mouse models, CQ sensitizes PDAC tumors to dual ICB (anti-PD-1 and anti-CTLA4 antibodies) (28). Moreover, vacuolar protein sorting 34 (Vps34) acts on the recruitment of autophagy-associated proteins and controls the formation of autophagosomes, research has demonstrated that the suppression of Vps34 enhances the efficacy of anti-PD-1/PD-L1 immunotherapy in preclinical models of melanoma and colorectal carcinoma (40). Nevertheless, in a C57BL/6J mouse model established by intradermal injection of B16-PD-L1 melanoma cells, Krueger et al. found that the autophagy inhibitor hydroxychloroquine (HCQ) impaired the efficacy of anti-PD-1 ICB therapy (41), suggesting that the efficacy of combining autophagy inhibitors with ICB therapy may be influenced by the specific TME associated with particular cancer types. On the other hand, in addition to employing autophagy inhibitors, promoting autophagy induction may similarly contribute to enhancing the therapeutic outcomes of ICB therapy. According to the study of human melanoma clinical samples and mouse models, the application of autophagy activator adiponectin ADIPOQ and 2-aminonicotinonitrile compound (w09) has potential therapeutic synergy with CTLA-4 inhibitors (42). These preclinical findings indicate that autophagy modulators hold potential for combination therapy with ICB therapy; however, their applicable scenarios require further exploration.

## Regulation of immune functions in innate immune cells

The innate immune cells in immunosuppressive TME mainly encompass tumor-associated macrophages (TAMs), tumor-associated neutrophils (TANs), dendritic cells (DCs), among others.

TAMs represent the most abundant innate immune population in TIME, which have two classical polarized phenotypes M1 and M2; M1 polarization has been verified to correlate with the tumoricidal function of TAMs that could engulf cancer cells and recruit T cells, while M2 polarization is associated with an immune suppressive profile (43). M2-polarized TAMs release cytokines such as interleukin-10 (IL-10) and transforming growth factor-β (TGF-β), exerting immunosuppressive effects through multiple mechanisms including suppressing the activation of B cells and T cells, impairing the maturation and functionality of antigen-presenting cells (APCs) like DCs, promoting CD8+ T cell exhaustion, and upregulating the expression of inhibitory immune checkpoint molecules (44). Previous studies have revealed that DDIT4-mediated inhibition of the PI3K-Akt/mTOR pathway promotes a metabolic shift toward oxidative phosphorylation while suppressing glycolysis. This reprogramming restricts macrophage polarization into the M1 phenotype, as immunostimulatory M1-type TAMs primarily rely on glycolysis for energy metabolism (45). Additionally, the activation of autophagy induced by DDIT4-mediated inhibition of mTOR is also observed in human decidual macrophages (dMφs); the pro-survival autophagy of dMφ can enhance the retention of immunosuppressive M2-type dMφ, while directly stimulating the invasion and proliferation of human choriocarcinoma cell line JEG3 (46). However, in both *in vitro* and *in vivo* models of breast cancer, Urolithin A has been shown to preserve the tumoricidal function of TAMs by inducing transcription factor EB (TFEB)-mediated mitophagy, partially via mTOR inhibition; this process concurrently decreases the M2-type TAM population within TIME (47). The discrepancy between the findings of these two studies may be attributed to the non-exclusive pathways involved in the activation of cellular autophagy. Furthermore, in bone marrow-derived macrophages, adipose tissue macrophages, and Kupffer cells, the mRNA-stabilizing factor methyltransferase-like 3 (METTL3), which promotes DDIT4 mRNA degradation through N6-methyladenosine (m6A), has also been shown to reduce the polarization of macrophages toward the M2 phenotype (48).

Regarding the regulation of DDIT4 on TANs, in NSCLC cell lines H-1299 and A549, DDIT4 facilitates the recruitment of TANs under hypoxic; these TANs, typically exhibit immunosuppressive properties in TME and can promote tumor growth, migration, and invasion (49). In neutrophils that have not yet differentiated into TANs, DDIT4 can promote the release of neutrophil extracellular traps (NETs) by mediating cellular autophagy (50). NETs are extracellular structures released by neutrophils, primarily composed of DNA, histones, antimicrobial peptides, and enzymes such as myeloperoxidase (MPO) and elastase, they play a crucial role in capturing and eliminating pathogens during infections; however, within the TIME, NETs also contribute to tumor progression and immune evasion by capturing and neutralizing immune cells (such as T cells and natural killer cells), promoting the infiltration of immunosuppressive cells (such as myeloid-derived suppressor cells and regulatory T cells), and releasing immunosuppressive cytokines (such as TGF-β and IL-10) (51). In a study focusing on patients with acute myeloid leukemia, NETs have been demonstrated to be associated with an immunosuppressive immune cell infiltration pattern and the high expression of inhibitory immune checkpoints within TME (52).

Other regulations of DDIT4 on innate immune system include effects on DCs. The maturation of DCs is essential for effective antigen presentation and T cell activation, however, DDIT4 overexpression impairs X-box binding protein 1 (XBP1) mRNA splicing, leading to DC reversion to an immature state and reduced antigen-presenting capacity (53). Additionally, rapamycin, which shares a consistent inhibitory effect on mTORC1 with DDIT4, has been demonstrated to induce the upregulation of immunoglobulin-like transcript 3(ILT3) and immunoglobulin-like transcript 4 (ILT4) on the surface of DCs, leading to an increase in the number of regulatory T cells (Tregs) as well as the expansion of the CD8(+)CD28(-) T cell subset deemed to be in a senescent state (54). This mechanism may potentially aid tumor cells in evading immune surveillance within the TIME. Moreover, DDIT4 upregulation has been observed to enhance the migratory and invasive potential of cervical cancer cells by activating the NF-κB pathway, a key regulator of innate immune responses (55).

In summary, the alterations in signaling pathways and metabolic states induced by high expression of DDIT4 exert a broadly suppressive regulatory effect on various components of the innate immune system, thus critically impairing adaptive immune activation as well.

## Regulation of immune functions in adaptive immune cells

The infiltration level of adaptive immune cells within TIME is strongly associated with tumor prognosis. T cells, as the primary effector cells of the adaptive immune system, play a crucial role in tumor immunity. Cytotoxic T lymphocytes (CTLs, CD8+ T cells) directly eliminate tumor cells by recognizing tumor antigen-MHC complexes and costimulatory signals presented on tumor cell surfaces via their T cell receptors (TCRs). Helper T cells (Ths, CD4+ T cells) enhance anti-tumor immunity through the secretion of cytokines such as IFN-γ and IL-2, which amplify immune responses. In contrast, Tregs facilitate tumor immune evasion by producing immunosuppressive factors like TGF-β and IL-10. B cells, another key component of TIME, function as APCs and differentiate into plasma cells that produce antibodies, while memory B cells provide long-term immunological memory, ensuring sustained protection against tumor recurrence (56).

The induction of autophagy activation through DDIT4-mediated suppression of mTORC1 represents a potential mechanism for the regulation of T cell function. A widely accepted view is that nearly all adaptive immune cells rely on pro-survival autophagy for the execution of their functions (57). Studies have shown that, significant autophagy associated protein Vps34 helps to maintain the viability and functionality of Tregs, which sustains an immunosuppressive environment (58); autophagy deficiency in invariant natural killer T (iNKT) cells leads to a reduction in the secretion of IL-4 and IFN-γ, which are key mediators of Th2 and Th1 immune responses, respectively (59); deletion of the autophagy-related protein phosphatidylinositol 3-kinase catalytic subunit type 3 (PIK3C3)/VPS34 reduced mitochondrial activity upon T cell activation, thereby inhibiting CD4+ T cell differentiation into Th1 cells (60).

However, beyond its pro-survival and functional maintenance roles, the activation of autophagy could also undermines the functional efficacy of T cells in the TIME via other underlying mechanisms. Concretely, autophagy in T cells with antitumor activity promotes the degradation of cytolytic granules produced by CD8+ T cells and natural killer (NK) cells, thereby impairing their tumor-killing capacity (61, 62); work by Mgrditchian et al. also demonstrated that targeted inhibition of the autophagy-related gene *Beclin1* in B16-F10 melanoma models, both *in vitro* and *in vivo*, significantly upregulates C-C motif chemokine ligand 5 (CCL5) expression, correlating with enhanced infiltration of NK cells and T cells into tumor tissues (63). Conversely, in a more recent study, researchers found that andrographolide (AD) induces autophagy, leading to increased CD8+ T cell infiltration in tumor tissues of AD-treated mice in both H1975 human NSCLC xenograft and Lewis lung carcinoma models; this effect was attributed to the more pronounced autophagic activity in tumor cells, where accelerated PD-L1 degradation alleviated the suppression of CD8+ T cells (64). These inconsistent findings further highlight the complexity of autophagy processes potentially induced by DDIT4 in the regulation of adaptive immune cells.

Aside from potentially regulating T cell function through autophagy activation, DDIT4-mediated inhibition of PI3K-Akt/mTOR pathway also modulates T cell activity by reprogramming metabolic states. Based on previous studies, mTOR regulates key transcription factors essential for coordinating glycolysis, including hypoxia-inducible factor 1-alpha (HIF-α) and myelocytomatosis oncogene (Myc); as well as fatty acid oxidation, mediated by peroxisome proliferator-activated receptor alpha (PPARα) and peroxisome proliferator-pctivated receptor gamma (PPARγ); in addition to fatty acid synthesis governed by sterol regulatory element-binding protein (SREBP); these transcription factors are also fundamentally involved in the processes of T-cell activation from a naive state, proliferation, antigen recognition, and subsequent functional responses (65). Generally, the inhibition of mTOR reduces the proliferation of adaptive immunocytes, thereby diminishing immune system activity; as a result, mTOR inhibitors are utilized as immunosuppressive agents in clinical practice (66). Furthermore, regarding the potential alteration of immunostimulatory and immunosuppresive T cell subsets caused by the active state of mTORC1, Zeng et al. has demonstrated that mTORC1 activation promotes immunosuppresive Treg proliferation and maintains Treg cell function stability through the facilitation of cholesterol/lipid metabolism (67). And in murine models of pancreatic cancer, IL-18-induced activation of the PI3K-Akt/mTOR pathway leads to the exhaustion of intratumoral CD8+ T cells (68), suggesting that mTOR inhibition mediated by DDIT4 may potentially ameliorate this process. The evidence provided by the aforementioned two studies partially supports that PI3K-Akt/mTOR pathway inhibition promotes T cell differentiation into a cytotoxic phenotype, further highlighting the complexity of DDIT-mediated mTORC1 inhibition in modulating cellular immune responses. Integrating the findings that DDIT4 inhibits PI3K-Akt/mTOR pathway to induce autophagy and that mTORC1 regulates T cell activation, proliferation, and differentiation through metabolic reprogramming, it is clear that DDIT4-mediated regulation of adaptive immune cell functions via the PI3K-Akt/mTOR pathway involves complex, interconnected mechanisms.

Additionally, consistent with the role of the mTORC1 as a critical downstream regulator of TCR signaling, B cell receptor (BCR) signaling also activates the downstream PI3K- Akt/mTOR signaling pathway, thus providing the energy required for the proliferation and activation of B cells (69). Regarding the probable impact of mTORC1 inhibition-induced autophagy on B cells in TIME, studies indicate that autophagy promotes B cell survival by clearing damaged organelles and protein aggregates, additionally, autophagy modulates antigen presentation capacity of B cells, thereby influencing T cell activation and antitumor immune responses (70). The more detailed regulatory mechanisms through which inhibitors such as DDIT4, suppress mTORC1 complex and subsequently modulate B cells to either enhance or suppress tumor immunity require further elucidation.

## Decoding regulatory dynamics through cellular crosstalk in TIME

Along with tumor cells and immune cells, other stromal cells residing within TIME also play a pivotal role in the regulation of tumor immunity, with fibroblasts serving as a crucial representative of this cellular constituency. The attenuation of mTORC1 activity induces intracellular metabolic reprogramming, manifesting as diminished glucose and glutamine metabolism concomitant with heightened ROS activity, which leads to the activation of cancer-associated fibroblasts (CAFs); the immunosuppressive phenotype of CAFs is marked by an augmented secretion of cytokines including IL-6 and TGF-β (71). As intercellular messengers, these cytokines can be received by both tumor cells and immune cells, thereby modulating their immune functions.

Collectively, the complicated regulation of the TIME involves the individual immunological functions of various cellular components, while the formation of a comprehensive immune regulatory network necessitates the interconnection of these individual immunological functions, wherein intercellular interactions mediated by secreted communicatory factors play an indispensable role. In addition to the immunosuppressive factors such as IL-10 and TGF-β secreted by previously mentioned M2-type TAMs and NETs, exosomes and senescence-associated secretory phenotypes (SASPs) released by cells also play significant roles in immunomodulatory cellular crosstalk.

Exosome biogenesis is a form of unconventional protein secretion mediated by the lysosomal system, exosomes derived from APCs carry diverse immune-related factors including immune-stimulating molecules such as MHC and co-stimulatory molecules, as well as immunosuppressive checkpoint molecules like PD-L1, thereby modulating immune cell activity through both activation and inhibition (24). Both autophagosomes and exosomes rely on intracellular multivesicular bodies (MVBs) to complete their respective functional pathways (72), this shared pathway component implies that DDIT4-mediated inhibition of the PI3K-Akt/mTOR pathway, which activates autophagy, may intricately regulate exosome secretion. Whether this results in increased or decreased exosome formation and release, as well as its impact on the immune microenvironment, is contingent on the specific cell type, tumor pathology, and the precise mechanism of autophagy activation.

DDIT4-mediated inhibition of mTORC1, which reduces endoplasmic reticulum (ER) biosynthesis, may induce ER stress, and apoptosis is triggered when this stress surpasses the regulatory capacity of senescent cells; within a certain cellular range, a decrease in senescent cell numbers leads to reduced secretion of SASPs, which constitute a collection of secretions from senescent cells comprising a variety of cytokines such as pro-inflammatory cytokines, growth factors, chemokines, and matrix-remodeling enzymes, these secretions play a role in inducing adjacent cells like recruiting immune cells and altering the phenotypic differentiation of immune cells (73). In both *in vitro* and *in vivo* models of glioblastoma, Peng et al. found that autophagy inhibition modifies the SASP profile secreted by senescent GBM cells, significantly enhancing anti-tumor immune responses in TIME, the underlying mechanisms involve promoting the polarization of TAMs towards the M1 phenotype and inhibiting the recruitment of TANs, and so on (74). These research findings indicate that the alterations in SASPs secretion and the consequent changes in intercellular communication signals induced by DDIT4-mediated inhibition of mTORC1 may play a non-negligible role in the regulation of the TIME.

**Figure 4:**
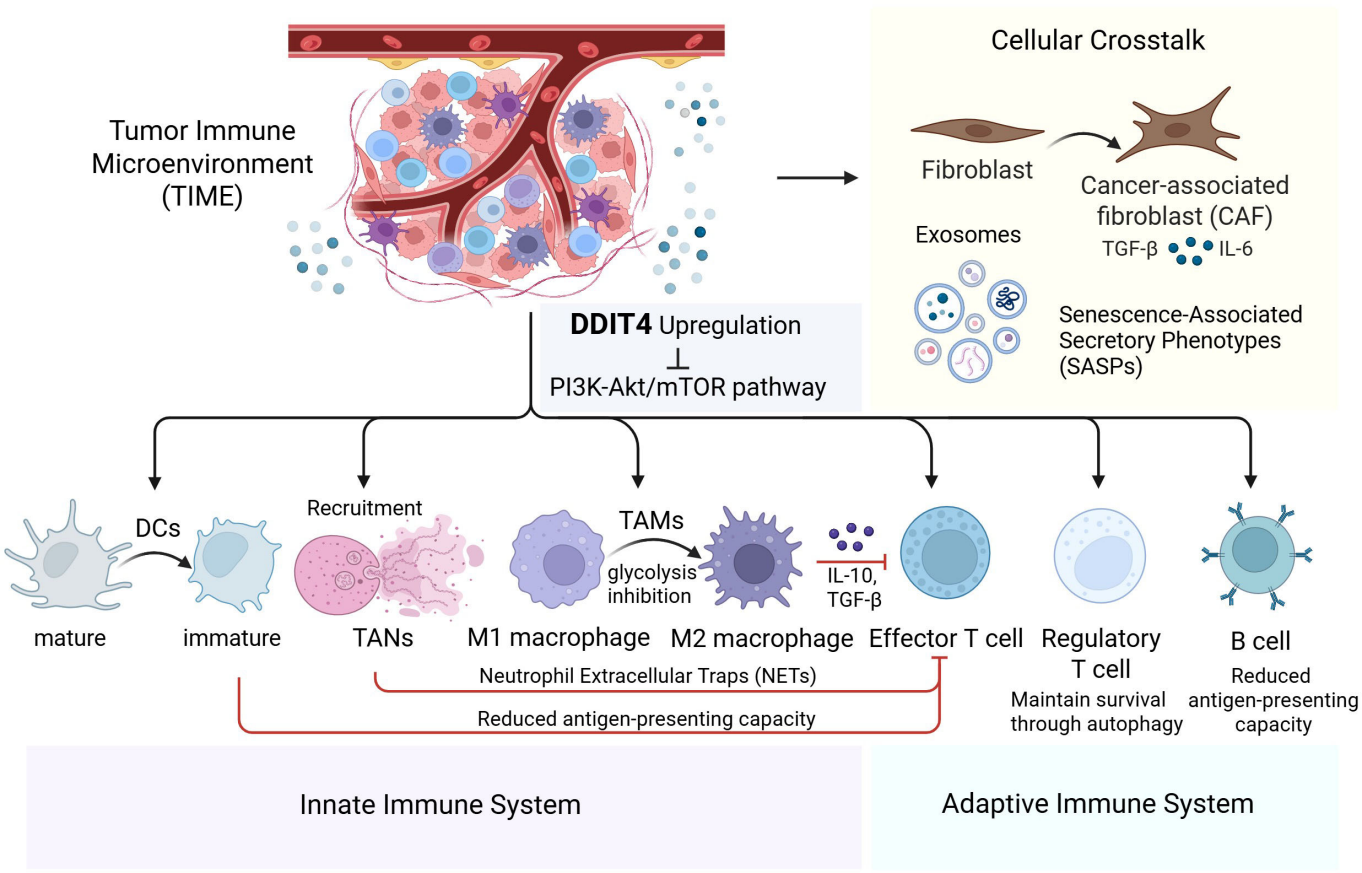
Comprehensive overview of DDIT4’s role in modulating innate/adaptive immunity and cellular crosstalk within the tumor immune microenvironment.

Overall, the alterations in the autophagy signaling pathway and cellular metabolic state induced by DDIT4-mediated inhibition of the PI3K-Akt/mTOR pathway may exert a pervasive influence on the diverse components of the intricate immune regulatory network within TIME (**Figure 4**).

This schematic depicts how DDT14 upregulation suppresses the PI3K-Akt/mTOR pathway, influencing both innate and adaptive immunity. In the innate immune system, DDT14 alters the functions of dendritic cells (DCs), tumor-associated neutrophils (TANs), and macrophages (TAMs). In the adaptive immune system, it modulates effector T cells, regulatory T cells, and B cells. Crosstalk with fibroblasts, cancer-associated fibroblasts (CAFs), exosomes, and senescence-associated secretory phenotypes (SASPs) further enhances immunosuppression, positioning DDT14 as a promising therapeutic target for cancer immunotherapy.

## Synergistic efficacy of autophagy modulators or mTOR inhibitors in combination with immunotherapy: clinical trial insights

As summarized above, the pathway in which DDIT4 inhibits the mTORC1 complex, thereby inducing downstream autophagy activation and metabolic reprogramming, plays a critical regulatory role in the TIME. Therapeutic strategies combining autophagy modulators or mTOR inhibitors with immunotherapy have been implemented in clinical trials targeting a variety of tumor types.

Hydroxychloroquine (HCQ), one of the most commonly used autophagy inhibitors in clinical trials, functions by inhibiting lysosomal acidification (75). In clinical trials investigating the combination of autophagy inhibitors with immunotherapy, clinical research UPCI 11-080 (NCT01550367) revealed that the combination of IL-2 and HCQ demonstrated good tolerability and clinical efficacy in treating metastatic renal cell carcinoma (RCC), with a notable increase in progression-free survival (PFS) exceeding 17 months at the 600 mg/d HCQ dose, representing a more than fourfold improvement compared to historical controls (76). While MEKiAUTO (NCT04214418) showed the combination of mitogen-activated protein kinase kinase inhibitor (MEKi) cobimetinib, HCQ, and PD-L1 inhibitor Atezolizumab demonstrated limited tolerability and efficacy in patients with advanced KRAS-mutated pancreatic ductal adenocarcinoma, clinical benefit was observed in 36% of patients, while all of whom achieved stable disease as their best response; and patients with KRAS^G12R^ mutations showed a trend toward improved overall survival (OS) compared to those with KRAS^non-G12R^ mutations (77). In addition, according to results of pre-operative trial (NCT03344172) targeting resectable pancreatic cancer posted on ClinicalTrials.gov, comparing the PGHA group using HCQ, gemcitabine, nab-paclitaxel and PD-L1 inhibitor Avelumab, with the PGH group using HCQ, gemcitabine and nab-paclitaxel, although the proportion of patients achieving grade IIb or higher histopathological responses was lower in the PGHA group (33.3%) compared to the PGH group (50.0%), the PGH group demonstrated a greater mean reduction in CA19-9 levels; and both treatment regimens showed limited safety profiles.

Furthermore, in addition to the aforementioned findings, several other clinical trials investigating the efficacy of combining autophagy modulators with immunotherapy (e.g., NCT04464759, NCT03754179, NCT05448677, NCT04841148, NCT04787991) are currently ongoing, with no results published to date.

A series of clinical trials have also explored the combination of mTOR inhibitors with immunotherapy as a therapeutic strategy for cancer. In the clinical trial (NCT03190174) investigating the combination of the mTOR inhibitor ABI-009 (Nab-rapamycin) with the PD-1 inhibitor Nivolumab for the treatment of various previously treated malignant sarcomas, the research has established the appropriate dosing regimen for the combination currently, which will be used as the recommended dose in further studies exploring disease control rate (DCR), PFS and OS (78). In addition, several other clinical trials investigating the combination of mTOR inhibitors and ICB therapy are currently ongoing, although no results have been published to date; these include: (1) Phase Ib/II non-randomized three-armed study (NCT02423954) investigated the efficacy of combining mTOR inhibitor Temsirolimus with Nivolumab in treating several types of advanced cancers; (2) Phase IIb randomized trial (NCT04203901) explored the efficacy of mTOR inhibitor Everolimus and tyrosine kinase inhibitor Lenvatinib combining Nivolumab and CTLA-4 inhibitor Ipilimumab in treating advanced RCC; (3) Phase II study (NCT05896839) investigated the efficacy of mTOR inhibitor Sirolimus and Prednisone combining Nivolumab and Ipilimumab in treating kidney transplant recipients with unresectable or metastatic skin cancer; (4) Phase Ib, open-label, multi-center study (NCT02890069) investigated the efficacy of using Everolimus and PD-1 inhibitor PDR001 (Spartalizumab) in combination in treating colorectal cancer, non-small cell lung carcinoma (adenocarcinoma), triple negative breast cancer and RCC; (5) Phase IIb study (NCT02430363) explored the efficacy of combining PI3K/Akt inhibitor NVP-BEZ235 with PD-1 inhibitor Pembrolizumab in treating glioblastoma (**Table 1**).

**Table 1:** Summary of clinical trials testing combinations of immunotherapy with autophagy modulators or mTOR inhibitors.

Combination Strategy	Tumor type	Phase	Enrollment	Treatment	Clinical Trial Identification Number
Combine immunotherapy with autophagy modulator	metastatic renal cell carcinoma	I/II	30	HCQ + Aldesleukin (IL-2)	NCT01550367
	gastrointestinal cancer	I/II	27	HCQ + Cobimetinib + Atezolizumab	NCT04214418
	resectable pancreatic cancer	II	32	HCQ + Gemcitabine + Nab-paclitaxel + Avelumab	NCT03344172
Combine immunotherapy with mTOR inhibitor	various malignant sarcomas	I/II	34	ABI-009 (Nab-rapamycin) + Nivolumab	NCT03190174
	several types of advanced cancers	Ib/II	33	Temsirolimus + Nivolumab	NCT02423954
	advanced renal cell carcinoma	IIb	16	Everolimus + Lenvatinib + Nivolumab + Ipilimumab	NCT04203901
	kidney transplant recipients with unresectable or metastatic skin cancer	II	16	Sirolimus + Prednisone + Nivolumab + Ipilimumab	NCT05896839
	colorectal cancer, non-small cell lung carcinoma (adenocarcinoma), triple negative breast cancer, renal cell carcinoma	Ib	298	Everolimus + PDR001	NCT02890069
	glioblastoma	IIb	58	NVP-BEZ235 + Pembrolizumab	NCT02430363

Taken togetehr, a series of clinical trials investigating the combination of autophagy modulators or mTOR inhibitors with immunotherapy are currently underway, and further clinical trial data as well as future clinical applications are highly anticipated.

## Discussion

DDIT4 is a regulatory factor that is commonly overexpressed in tumor tissues. Its inhibitory effect on mTORC1 can lead to dysfunction in the PI3K-Akt/mTOR pathway, which typically promotes anabolic metabolism and cell proliferation, thereby triggering various effects such as intracellular metabolic reprogramming and activation of autophagy. This review highlights the impact of these effects on TIME, aiming to provide evidence supporting DDIT4 as a novel target for tumor immunotherapy.

This review separately analyzes the regulation of immune function in different cellular components of the TIME by mTOR inhibition-induced autophagy activation or metabolic reprogramming, and crosstalk between them as well. However, in light of prior research, the current conclusions presented herein possess inherent limitations. First, the heterogeneity in TIME functional changes induced by DDIT4 overexpression across different tumor types remains inadequately explained, necessitating further analysis based on the biochemical or pathological characteristics of specific tumors. Second, in specific cellular components of the TIME, the regulation of immune function by DDIT4-mediated inhibition of the PI3K-Akt/mTOR pathway is a combined effect of multiple mechanisms (e.g., changes in metabolic state and autophagy activation); and whether to promote anti-tumor immunity or facilitate tumor immune evasion, there are varying changes among different cell types. Finally, given that the TME is a complex ecosystem composed of various cellular and non-cellular components, the expression and functional levels of DDIT4 in different cell types, as well as its overall contribution to tumor immunity, require further clarification. To address these limitations, further in-depth research into the molecular mechanisms is required in the future.

Despite these limitations, this review demonstrates that DDIT4, through its inhibition of the PI3K-Akt/mTOR pathway, exerts widespread and profound regulatory effects on various components of the TIME, highlighting its potential as a target for developing novel immunotherapies and overcoming immunotherapy resistance. This underscores its significance as a crucial element in the future development of new combination therapies and targeted drugs.
