# Correction: A review of the participation of DDIT4 in the tumor immune microenvironment through inhibiting PI3K-Akt/mTOR pathway

**DOI:** 10.3389/fonc.2025.1693680

**Published:** 2025-09-04

**Authors:** Yunshu Jiao, Yang Xiang

**Affiliations:** ^1^ National Clinical Research Center for Obstetric & Gynecologic Diseases, Department of Obstetrics and Gynecology, Peking Union Medical College Hospital, Chinese Academy of Medical Sciences & Peking Union Medical College, Beijing, China; ^2^ Clinical College, Chinese Academy of Medical Sciences and Peking Union Medical College, Beijing, China

**Keywords:** DDIT4, mTORC1, tumor immune microenvironment, autophagy, PI3K-Akt/mTOR pathway

Affiliation “National Clinical Research Center for Obstetric & Gynecologic Diseases, Department of Obstetrics and Gynecology, Peking Union Medical College Hospital, Chinese Academy of Medical Sciences & Peking Union Medical College, Beijing, China” was erroneously given as “Department of Obstetrics and Gynecology, Peking Union Medical College Hospital, Chinese Academy of Medical Sciences and Peking Union Medical College, Beijing, China”.

Affiliation “National Clinical Research Center for Obstetric & Gynecologic Diseases, Department of Obstetrics and Gynecology, Peking Union Medical College Hospital, Chinese Academy of Medical Sciences & Peking Union Medical College, Beijing, China” was omitted for author Yunshu Jiao. This affiliation has now been added for author Yunshu Jiao.

The original version of this article has been updated.

