# Supplementary material for: A review of the participation of DDIT4 in the tumor immune microenvironment through inhibiting PI3K-Akt/mTOR pathway
Source: Front Oncol. 2025 Aug 18;15:1595463. doi: 10.3389/fonc.2025.1595463 (PMC12399382; doi:10.3389/fonc.2025.1595463)
Supplement: Supplementary file 1 [file Table1.docx]

**Supplementary Table S1. Functions of DDIT4 Across Various Cancer Types**

|  | Cancer Types | DDIT4 functions | References |
| --- | --- | --- | --- |
| In vitro model | Non-Small Cell Lung Cancer | Negative regulation of invasion of lung cancer cells. | (1) |
|  | Breast Cancer | Acts as oxidative stress regulator and contributes to arsenic trioxide-induced apoptosis in cancer cells. | (2) |
|  | Pancreatic Cancer | Downregulation of DDIT4 sustains cancer cell stemness and chemoresistance. | (3) |
|  | Anaplastic Thyroid Cancer | Degradation of DDIT4 induces activation of ^1^EMT and promotes tumor progression. | (4) |
|  | Esophageal Squamous Cell Carcinoma | DDIT4 expression enhanced by romidepsin inhibits cancer cell proliferation. | (5) |
|  | Gastric Cancer | Promotes cancer cell proliferation and tumorigenesis. | (6) |
|  | Cervical Cancer | Knockdown of DDIT4 inhibits cell proliferation and migration, while inducing apoptosis. | (7) |
|  | Glioblastoma Multiforme | Up-regulation of DDIT4 leads to desensitization against temozolomide cytotoxicity in cancer cells. | (8) |
|  | Triple-Negative Breast Cancer | DDIT4 expression negatively correlates with immune infiltration, but positively with immune checkpoints in cancer tissues. | (9) |
|  | Lung Adenocarcinoma | Up-regulation of DDIT4 enhances the migration and invasion of A549 cells; High-DDIT4 tumors exhibit increased M2 macrophage infiltration and elevated expression of immune checkpoint genes including PD-L1 and PD-1. | (10, 11) |
|  | Nasopharyngeal Carcinoma | DDIT4 expression promoted by HIF1α stimulates cancer cell proliferation, invasion, migration, and inhibits apoptosis. | (12) |
|  | Renal Cell Carcinoma | DDIT4 determines cancer cell responsiveness to autophagy inhibitors like chloroquine. | (13) |
|  | Acute Myeloid Leukemia | Knockdown of DDIT4 suppresses cancer cell proliferation while inducing apoptosis and cell cycle arrest. | (14) |
|  | Oral Squamous Cell Carcinoma | DDIT4 suppression enhances cisplatin sensitivity in cancer cells. | (15) |
| Clinical relevance  analysis | Endometrial Cancer | High expression correlates to favorable prognosis including ^2^PFS and ^3^OS. | (16) |
|  | Colorectal Cancer | Overexpression is related to advanced pathological features of patients. | (17) |
|  | Prostate Cancer | DDIT4 considerably elevates in patients with bone metastasis. | (18) |
|  | Lung Adenocarcinoma | Serves as an independent prognostic factor for shorter OS in patients. | (19) |
|  | Head and Neck Squamous Cell Carcinoma | Upregulation positively correlates with larger tumor size, lymph node metastasis, more advanced ^4^TNM stage and higher tumor mutational burden, while negatively correlates with immune score. | (20) |
|  | Acute Myeloid Leukemia | High DDIT4 expression serves as a poor prognostic factor. | (21) |
|  | Pancreatic Cancer | Nuclear overexpression of DDIT4 associates with advanced TNM stage and lymphovascular invasion in ^5^PDAC and ^6^PNET. | (22) |

^1^EMT: Epithelial-Mesenchymal Transition; ^2^PFS: Progression-Free Survival; ^3^OS: Overall Survival; ^4^TNM: Tumor-Node-Metastasis; ^5^PDAC: pancreatic ductal adenocarcinoma; ^6^PNET: pancreatic neuroendocrine tumor;

**Supplementary Table S2.** **Impact of DDIT4-Mediated PI3K-Akt/mTOR Pathway Inhibition on Tumor Cell Immune Checkpoint Expression and the Efficacy of Combining Autophagy Modulators with ^1^ICB Therapy in Preclinical Cancer Models**

|  | Cancer Types | Effect | References |
| --- | --- | --- | --- |
| DDIT4-mTOR-autophagy axis regulates immune checkpoint expression | Non-Small Cell Lung Cancer | The activation of p62-dependent autophagy by DDIT4 may lead to reduced PD-L1 expression. | (23, 24) |
|  | Glioblastoma | DDIT4-activated autophagy may suppress STAT3 phosphorylation, thereby inhibiting transcriptional activation of the PD-L1 gene. | (25) |
|  | Osteosarcoma | Autophagy inhibitor 3MA suppresses PD-L1 expression. | (26) |
|  | Triple-Negative Breast Cancer | DDIT4 expression positively correlates with immune checkpoint molecules, including PD-L1 and CTLA4. | (9) |
|  | Lung Adenocarcinoma | High-DDIT4 tumors exhibit elevated expression of immune checkpoint genes including PD-L1 and PD-1. | (11) |
| Efficacy of combining autophagy modulators with ICB therapy in preclinical research | Pancreatic Ductal Adenocarcinoma | Autophagy inhibitor ^2^CQ sensitizes tumors to dual ICB (anti-PD-1 and anti-CTLA4 antibodies). | (27) |
|  | Melanoma and Colorectal Carcinoma | Suppression of Vps34-mediated autophagosome initiation enhances anti-PD-1/PD-L1 therapeutic effects. | (28) |
|  | Melanoma | Autophagy inhibitor ^3^HCQ diminishes the efficacy of anti-PD-1 ICB therapy. | (29) |
|  | Melanoma | Autophagy activation exhibits therapeutic synergy with CTLA-4 inhibition. | (30) |

^1^ICB: Immune Checkpoint Blockade; ^2^CQ: Chloroquine; ^3^HCQ: Hydroxychloroquine.

1. Bordoloi D, Padmavathi G, Banik K, Devi KA, Harsha C, Girisa S, et al. Human Tumor Necrosis Factor Alpha-Induced Protein Eight-Like 1 Exhibited Potent Anti-Tumor Effect through Modulation of Proliferation, Survival, Migration and Invasion of Lung Cancer Cells. *Mol Cell Biochem* (2021) 476(9):3303-18. Epub 2021/04/26. doi: 10.1007/s11010-021-04060-1.

2. Glorieux C, Calderon PB. Catalase down-Regulation in Cancer Cells Exposed to Arsenic Trioxide Is Involved in Their Increased Sensitivity to a Pro-Oxidant Treatment. *Cancer Cell Int* (2018) 18:24. Epub 2018/02/23. doi: 10.1186/s12935-018-0524-0.

3. Zhang Y, Liu X, Wang Y, Lai S, Wang Z, Yang Y, et al. The M(6)a Demethylase Alkbh5-Mediated Upregulation of Ddit4-As1 Maintains Pancreatic Cancer Stemness and Suppresses Chemosensitivity by Activating the Mtor Pathway. *Mol Cancer* (2022) 21(1):174. Epub 20220902. doi: 10.1186/s12943-022-01647-0.

4. Dai B, Xu L, Rong S, Song M, Lan Z, Chen W, et al. Ythdf2 Promotes Anaplastic Thyroid Cancer Progression by Activating the Ddit4/Akt/Mtor Signaling Pathway. *Biol Direct* (2024) 19(1):122. Epub 20241126. doi: 10.1186/s13062-024-00566-y.

5. Xia WF, Zheng XL, Liu WY, Huang YT, Wen CJ, Zhou HH, et al. Romidepsin Exhibits Anti-Esophageal Squamous Cell Carcinoma Activity through the Ddit4-Mtorc1 Pathway. *Cancer Gene Ther* (2024) 31(5):778-89. Epub 20240313. doi: 10.1038/s41417-024-00760-0.

6. Du F, Sun L, Chu Y, Li T, Lei C, Wang X, et al. Ddit4 Promotes Gastric Cancer Proliferation and Tumorigenesis through the P53 and Mapk Pathways. *Cancer Commun (Lond)* (2018) 38(1):45. Epub 2018/07/07. doi: 10.1186/s40880-018-0315-y.

7. Su J, Zhao Y, Chen WD, Wang YD. Activation of the G Protein-Coupled Bile Acid Receptor Tgr5 Modulates the Hcp5/Mir-139-5p/Ddit4 Axis to Antagonize Cervical Cancer Progression. *Int J Mol Sci* (2024) 25(16). Epub 20240816. doi: 10.3390/ijms25168932.

8. Ho KH, Chen PH, Chou CM, Shih CM, Lee YT, Cheng CH, et al. A Key Role of DNA Damage-Inducible Transcript 4 (Ddit4) Connects Autophagy and Glut3-Mediated Stemness to Desensitize Temozolomide Efficacy in Glioblastomas. *Neurotherapeutics* (2020) 17(3):1212-27. doi: 10.1007/s13311-019-00826-0.

9. Chen X, Li Z, Liang M, Zhang Z, Zhu D, Lin B, et al. Identification of Ddit4 as a Potential Prognostic Marker Associated with Chemotherapeutic and Immunotherapeutic Response in Triple-Negative Breast Cancer. *World J Surg Oncol* (2023) 21(1):194. Epub 20230630. doi: 10.1186/s12957-023-03078-7.

10. Song X, Liu B, Zhao G, Pu X, Liu B, Ding M, et al. Streptococcus Pneumoniae Promotes Migration and Invasion of A549 Cells in Vitro by Activating Mtorc2/Akt through up-Regulation of Ddit4 Expression. *Front Microbiol* (2022) 13:1046226. Epub 20221219. doi: 10.3389/fmicb.2022.1046226.

11. Hua X, Wang Y, Wang C, Yang H, Liu A. Hypoxia-Related Gene Ddit4 as a Therapeutic Biomarker Promotes Epithelial-Mesenchymal Transition in Lung Adenocarcinoma Via the Mapk/Erk Signaling Pathway. *Int Immunopharmacol* (2025) 157:114739. Epub 20250501. doi: 10.1016/j.intimp.2025.114739.

12. Zhao J, Li B, Ren Y, Liang T, Wang J, Zhai S, et al. Histone Demethylase Kdm4a Plays an Oncogenic Role in Nasopharyngeal Carcinoma by Promoting Cell Migration and Invasion. *Exp Mol Med* (2021) 53(8):1207-17. Epub 20210812. doi: 10.1038/s12276-021-00657-0.

13. Carew JS, Espitia CM, Sureshkumar S, Carrera Espinoza MJ, Gamble ME, Wang W, et al. Redd1 Is a Determinant of the Sensitivity of Renal Cell Carcinoma Cells to Autophagy Inhibition That Can Be Therapeutically Exploited by Targeting Pim Activity. *Cancer Lett* (2025) 613:217496. Epub 20250130. doi: 10.1016/j.canlet.2025.217496.

14. Wang M, Xian H, Xia X, Zhang W, Huang Z, Lu C, et al. Establishment of a Prognostic Model Based on Er Stress-Related Cell Death Genes and Proposing a Novel Combination Therapy in Acute Myeloid Leukemia. *J Transl Med* (2025) 23(1):566. Epub 20250521. doi: 10.1186/s12967-025-06615-y.

15. Feng Y, Cao X, Zhao B, Song C, Pang B, Hu L, et al. Nitrate Increases Cisplatin Chemosensitivity of Oral Squamous Cell Carcinoma Via Redd1/Akt Signaling Pathway. *Sci China Life Sci* (2021) 64(11):1814-28. Epub 20210917. doi: 10.1007/s11427-020-1978-4.

16. Yoshikawa N, Yoshida K, Liu W, Matsukawa T, Hattori S, Yoshihara M, et al. The Prognostic Significance of Ddit4 in Endometrial Cancer. *Cancer Biomark* (2023) 37(4):217-25. doi: 10.3233/cbm-220368.

17. Fattahi F, Saeednejad Zanjani L, Habibi Shams Z, Kiani J, Mehrazma M, Najafi M, et al. High Expression of DNA Damage-Inducible Transcript 4 (Ddit4) Is Associated with Advanced Pathological Features in the Patients with Colorectal Cancer. *Sci Rep* (2021) 11(1):13626. Epub 2021/07/03. doi: 10.1038/s41598-021-92720-z.

18. Zhao Y, Hu X, Yu H, Sun H, Zhang L, Shao C. The Fto Mediated N6-Methyladenosine Modification of Ddit4 Regulation with Tumorigenesis and Metastasis in Prostate Cancer. *Research (Wash D C)* (2024) 7:0313. Epub 20240221. doi: 10.34133/research.0313.

19. Song L, Chen Z, Zhang M, Zhang M, Lu X, Li C, et al. Ddit4 Overexpression Associates with Poor Prognosis in Lung Adenocarcinoma. *J Cancer* (2021) 12(21):6422-8. Epub 20210903. doi: 10.7150/jca.60118.

20. Zhang Z, Zhu H, Zhao C, Liu D, Luo J, Ying Y, et al. Ddit4 Promotes Malignancy of Head and Neck Squamous Cell Carcinoma. *Mol Carcinog* (2023) 62(3):332-47. Epub 20221201. doi: 10.1002/mc.23489.

21. Cheng Z, Dai Y, Pang Y, Jiao Y, Liu Y, Cui L, et al. Up-Regulation of Ddit4 Predicts Poor Prognosis in Acute Myeloid Leukaemia. *J Cell Mol Med* (2020) 24(1):1067-75. Epub 20191121. doi: 10.1111/jcmm.14831.

22. Tajik F, Fattahi F, Rezagholizadeh F, Bouzari B, Babaheidarian P, Baghai Wadji M, et al. Nuclear Overexpression of DNA Damage-Inducible Transcript 4 (Ddit4) Is Associated with Aggressive Tumor Behavior in Patients with Pancreatic Tumors. *Sci Rep* (2023) 13(1):19403. Epub 20231108. doi: 10.1038/s41598-023-46484-3.

23. Liao H, Chang X, Gao L, Ye C, Qiao Y, Xie L, et al. Il-17a Promotes Tumorigenesis and Upregulates Pd-L1 Expression in Non-Small Cell Lung Cancer. *J Transl Med* (2023) 21(1):828. Epub 2023/11/18. doi: 10.1186/s12967-023-04365-3.

24. Huang X, Yao J, Liu L, Chen J, Mei L, Huangfu J, et al. S-Acylation of P62 Promotes P62 Droplet Recruitment into Autophagosomes in Mammalian Autophagy. *Mol Cell* (2023) 83(19):3485-501.e11. Epub 2023/10/07. doi: 10.1016/j.molcel.2023.09.004.

25. Tong L, Li J, Li Q, Wang X, Medikonda R, Zhao T, et al. Act001 Reduces the Expression of Pd-L1 by Inhibiting the Phosphorylation of Stat3 in Glioblastoma. *Theranostics* (2020) 10(13):5943-56. Epub 2020/06/03. doi: 10.7150/thno.41498.

26. Yu W, Wang Y, Zhu J, Jin L, Liu B, Xia K, et al. Autophagy Inhibitor Enhance Znpc/Bsa Nanoparticle Induced Photodynamic Therapy by Suppressing Pd-L1 Expression in Osteosarcoma Immunotherapy. *Biomaterials* (2019) 192:128-39. Epub 2018/11/19. doi: 10.1016/j.biomaterials.2018.11.019.

27. Yamamoto K, Venida A, Yano J, Biancur DE, Kakiuchi M, Gupta S, et al. Autophagy Promotes Immune Evasion of Pancreatic Cancer by Degrading Mhc-I. *Nature* (2020) 581(7806):100-5. Epub 2020/05/08. doi: 10.1038/s41586-020-2229-5.

28. Noman MZ, Parpal S, Van Moer K, Xiao M, Yu Y, Viklund J, et al. Inhibition of Vps34 Reprograms Cold into Hot Inflamed Tumors and Improves Anti-Pd-1/Pd-L1 Immunotherapy. *Sci Adv* (2020) 6(18):eaax7881. Epub 2020/06/05. doi: 10.1126/sciadv.aax7881.

29. Krueger J, Santinon F, Kazanova A, Issa ME, Larrivee B, Kremer R, et al. Hydroxychloroquine (Hcq) Decreases the Benefit of Anti-Pd-1 Immune Checkpoint Blockade in Tumor Immunotherapy. *PLoS One* (2021) 16(6):e0251731. Epub 20210628. doi: 10.1371/journal.pone.0251731.

30. Shukla SA, Bachireddy P, Schilling B, Galonska C, Zhan Q, Bango C, et al. Cancer-Germline Antigen Expression Discriminates Clinical Outcome to Ctla-4 Blockade. *Cell* (2018) 173(3):624-33.e8. Epub 2018/04/17. doi: 10.1016/j.cell.2018.03.026.
